# Data on the current state of modular systems in a highly dynamic environment: Empirical analyses in the manufacturing industry and automotive industry of Germany

**DOI:** 10.1016/j.dib.2019.104552

**Published:** 2019-10-08

**Authors:** Peter Burggräf, Matthias Dannapfel, Fabian Hehl, Miriam Wenzl, Bernhard Freyer

**Affiliations:** a University of Siegen, Germany; b RWTH Aachen University, Germany; c Friedrich-Alexander University Erlangen-Nuremberg, Germany; d Technical University of Munich, Germany

**Keywords:** Modular systems, Highly dynamic environment, Future boundary conditions, Innovations, Urgency, Product development

## Abstract

Today, many companies develop modular systems to realise considerable product differentiation and variation while simultaneously reducing costs through economies of scale [1]. Designing these modular systems to be lasting and robust in a highly dynamic environment [2], avoiding subsequent modification cost [3,4] and staying innovative over the product lifecycle is crucial for sustainable success in an increasingly competitive market [5]. Two closely related surveys were carried out on the initial situation described above. The first survey deals with the major conflict between planning reliability and flexibility with regard to future boundary conditions that the described challenges lead to. The data presented in this article on the first survey was collected from German companies, mainly from the automotive industry, developing and manufacturing complex products using modular systems. Other represented companies operate in the machine and plant construction industry. The survey comprises the answers of 39 participants, gathered via online questionnaire. The 17 questions of the survey are divided into two topics: characteristics of the participants, and the problem description of modular systems in highly dynamic environments and the management thereof. The second survey deals with the anticipation of innovations in the design of modular systems and the selection of the right choice from a variety of possible innovations to be considered. The data presented in this article on the second survey was collected from German automotive manufacturers, which develop and manufacture complex products using modular systems. The survey comprises the answers of 501 participants, gathered via online questionnaire. The 14 questions of the survey are divided into two topics: characteristics of the participants, and the problem description of modular systems and the management thereof. The data obtained allows the identification of existing deficits and dedicated research on solutions.

**Table:** Specifications Table

Subject	Engineering (General), Product development
Specific subject area	Modular systems and the management thereof
Type of data	Tables, figures
How data were acquired	Consultation of relevant participants and survey via online questionnaire. Questions and prescribed answer options are part of the attached data.
Data format	Raw, analyzed
Parameters for data collection	Survey 1: Data of 39 respondents covering 17 questions on modular systems in a highly dynamic environment. Survey 2: Data of 501 respondents covering 14 questions on modular systems.
Description of data collection	Survey 1: German companies that develop and manufacture complex products using modular systems (cf. automotive manufacturers, automotive suppliers, mechanical and plant engineering). Respondents who are responsible or involved in the development process of modular systems. Survey 2: German automotive manufacturers which develop and manufacture complex products using modular systems. Respondents who are responsible or involved in the development process of modular systems.
Data source location	Germany
Data accessibility	With the article - A comprehensive dataset is attached to this data article as a Microsoft Excel spreadsheet.
Related research article	G. Schuh, W. Schultze, M. Schiffer, A. Rieger, S. Rudolf, H. Lehbrink, Scenario-based determination of product feature uncertainties for robust product architectures, Production Engineering, 8 (3), 2014, 383–395. DOI: 10.1007/s11740-014-0532-4 [4]

**Table:** 

**Value of the Data** • While existing approaches in the literature on modular systems are predominantly concerned with the end product or with the question of which components can be optimally used as modular systems under certain conditions, the presented data allows an insight into the rather uninvestigated field of dealing with modular systems in a highly dynamic environment in the context of product development and securing innovation capability over the entire product life cycle in the context of product development. • The raw data enable additional analysis about the current state of modular systems in a highly dynamic environment and dealing with innovations. • The data describes the current status quo of modular systems in a highly dynamic environment and dealing with innovations in practice. Used as an explanation component, the data allows to reveal deficits and opportunities which may be addressed by dedicated research.

## 1 Data

### 1.1 Survey 1 – modular systems in a highly dynamic environment in the context of product development

The survey was conducted to confirm the specified need for action for modular systems in a highly dynamic environment in practice and to validate the solution approaches described. From November 2017 to March 2018, 39 Participants from numerous internationally active companies from Germany were interviewed 17 questions in an online questionnaire against the background of several hypotheses. The gathered data of the survey is divided into two consecutive modules. In the first part, the sample of the survey is presented and characterised. The second module deals with the problem description of modular systems. The data predominantly consists of ordinally scaled variables, either representing the participants' degree of consent to a statement, or a self-assessment of their situation and capabilities. The full dataset (Q1-17) is provided as supplementary material in [dataset] Appendix A.

### 1.2 Survey 2 – modular systems dealing with innovations

The survey was conducted to determine the status quo of modular systems in dealing with innovations and the specified need for action in practice. From May 2017 to July 2017, 501 participants from internationally active German automotive manufacturers were interviewed using 14 questions in an online questionnaire against the background of several hypotheses. The survey data collected is divided into two successive modules. In the first part, the sample of the survey is presented and characterized. The second module deals with the problem description of modular systems. The data consist predominantly of ordinal-scaled variables that represent either the degree of agreement of participants to a statement or a self-assessment of their situation and capabilities. The full dataset (Q1-Q14) is provided as supplementary material in [dataset] Appendix B.

## 2 Experimental design, materials, and methods

### 2.1 Survey 1 – modular systems in a highly dynamic environment in the context of product development

#### 2.1.1 Presentation of the survey sample

**Fig. 1:**
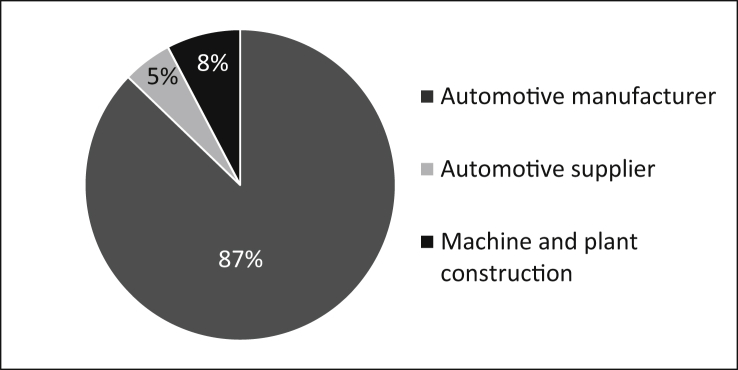
Represented industries.

The first part of the questionnaire is intended to characterise the sample of the survey. As the data in Fig. 1 shows most of the participants are active in the automotive industry (original equipment manufacturers and suppliers). Other participants work for companies in the mechanical and plant construction industry.

**Fig. 2:**
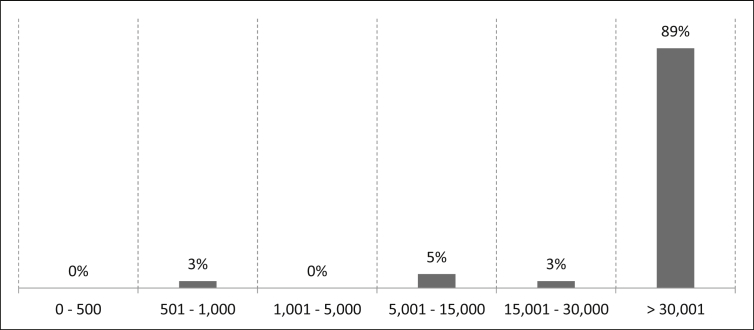
Number of staff [FTE] in the entire company.

On average, more than 30,001 people are employed in the companies in which the participants work (See Fig. 2). On the basis of the recommendation of the European Commission, all participating companies can be classified as large enterprises due to their number of employees (≥250) [6].

**Fig. 3:**
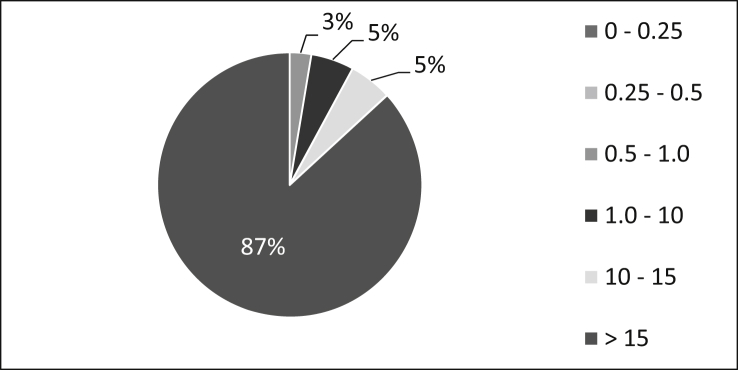
Annual turnover (in billions of €).

In order to be able to assess the economic situation of the participating companies, the annual turnover was used as a benchmark. As can be seen in Fig. 3 three percent of the participants state an annual turnover up to 0.25 billion €, five percent between 0.5 and 1.0 billion €, another five percent between 1.0 and 10 billion € and 87% above 15 billion €. These figures show that, according to the European Commission's definition, all companies in which the survey participants work are large companies with an annual turnover of more than 50 million € [6].

**Fig. 4:**
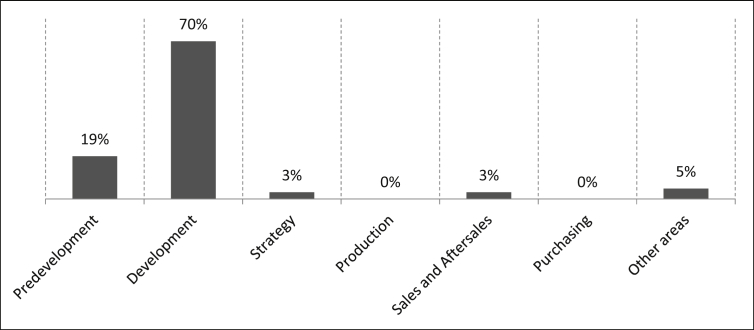
Business areas of the staff.

The business areas in which the survey participants work are as follows: 19% of the participants work in predevelopment, 70% in development, three percent in strategy and three percent in sales and after-sales. Another five percent are listed under the term “Other areas” and an example of this would be data and product management, as can be seen in Fig. 4.

**Fig. 5:**
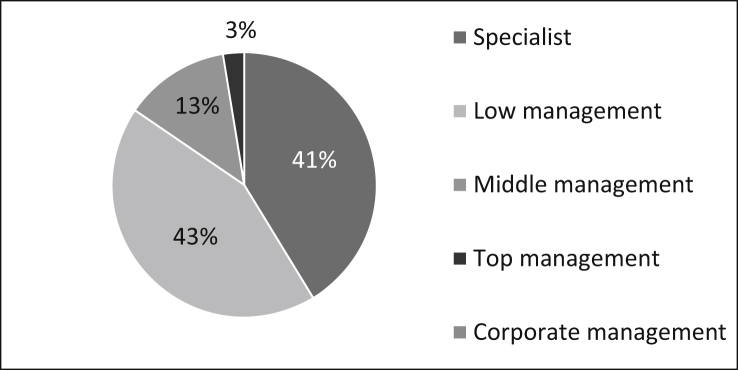
Roles of the staff.

The roles of the survey participants are manifold: 41% of the survey participants are specialists, 43% are active in lower management, 13% in middle management and three percent in top management (See Fig. 5).

**Fig. 6:**
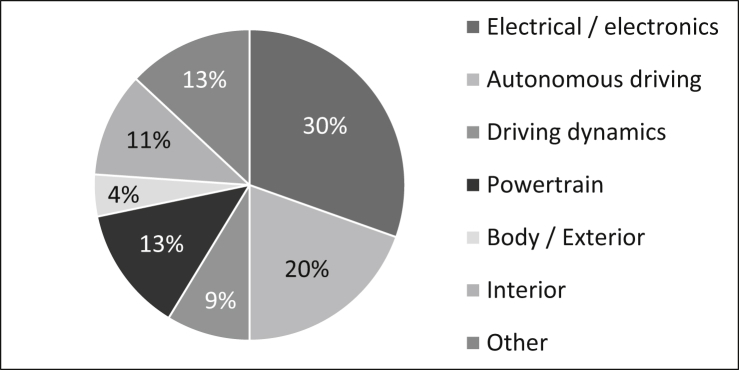
Subject areas of the staff.

The survey participants come from the following areas, as shown in Fig. 6: 30% work in the area of electrical/electronics, 20% in the area of autonomous driving, nine percent in the area of driving dynamics, 13% in the area of powertrain, four percent in the area of body/exterior, eleven percent in the area of interior and 13% in other areas such as plant engineering or validation planning. As the survey has a strong focus on the automotive industry, participants from the non-automotive industries in particular are listed in the group “Other”.

#### 2.1.2 Current situation of modular systems

In the second part of the survey, the participants are confronted with various statements as to whether and where companies see challenges through modular systems. Further statements deal in particular with the current situation and the challenges for modular systems in a highly dynamic environment. The number of samples for all studies is n = 39, unless otherwise specified.

**Fig. 7:**
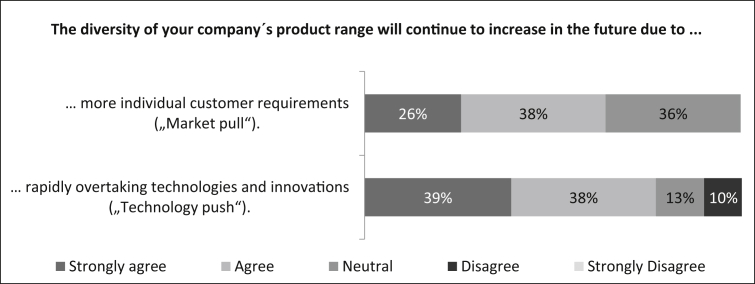
Reasons for increasing product diversity.

In the beginning, the participants are asked to rate their approval of two statements regarding the diversity of the product range. First they were asked if *“The diversity of your company's product range will continue to increase in the future due to more individual customer requirements (market pull).”* In Fig. 7 it can be seen that the majority (64%) of the survey participants agree or strongly agree with this statement. The remaining 36% rate the statement neutrally. Second they were asked if *“The diversity of your company's product range will continue to increase in the future due to rapidly overtaking technologies and innovations (technology push).”* The majority (77%) of participants agree with this statement, 13% rate this statement neutrally and only 10% disagree.

In the next question, the participants are confronted with two statements as to whether the economies of scale generated by modular systems achieve economic advantages on both the process and product sides.

**Fig. 8:**
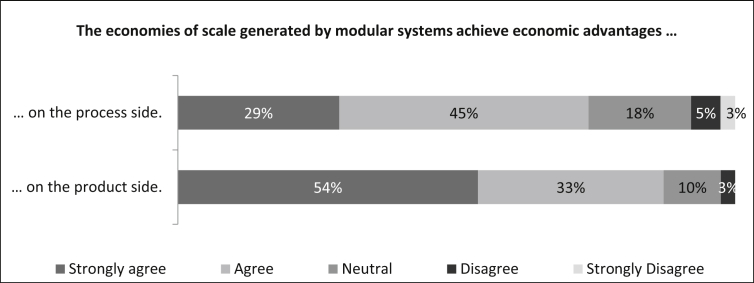
Generation of economic advantages through economies of scale.

As can be seen in Fig. 8, 74% of the participants agree or strongly agree that on the process side economic advantages are generated by the economies of scale of modular systems and the neutral evaluation is 18%. On the product side, there is an even greater approval rating of 87% and a lower neutral evaluation of 10%. Only a small minority of eight percent on the process side and three percent on the product side do not agree with the statements. The number of samples on the process side is n = 38. In combination with Fig. 7, this assessment confirms BARBOSA ET AL. statement that modular systems achieve significant product differentiation and variation while simultaneously reducing costs through economies of scale [1].

**Fig. 9:**
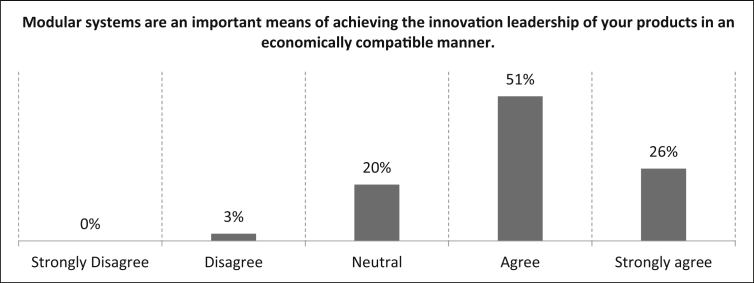
Evaluation of the achievement of innovation leadership by means of modular systems.

As already described in the introduction, the literature states that innovation is crucial for the company's success in securing the competitiveness of its own products [5]. Therefore the participants are asked for their degree of agreement with the statement *“Modular systems are an important means of achieving the innovation leadership of your products in an economically compatible manner*." in Fig. 9.

The vast majority (77%) of the participants confirm that the innovation leadership of their products can be achieved in an economically compatible manner by modular systems (26% strongly). There is a 20% rating on the neutral statement and only three percent disagree with the statement.

**Fig. 10:**
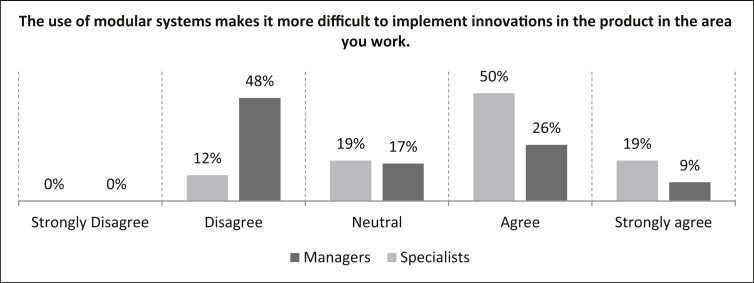
Rating of difficulties in the implementation of innovations through modular systems.

Next, the online questionnaire presented the proposition *“The use of modular systems makes it more difficult to implement innovations in the product in the area in which you work.”* and there are significant differences in the evaluation between specialists and managers, as described in the paragraph below (See Fig. 10).

With an agreement of 69%, specialists confirm the statement in the comparison to managers (35%). With a percentage of 48%, managers disagree more with the statement than the specialists with 12%. The neutral evaluation is at a nearly similar percentage level for both comparison groups.

**Fig. 11:**
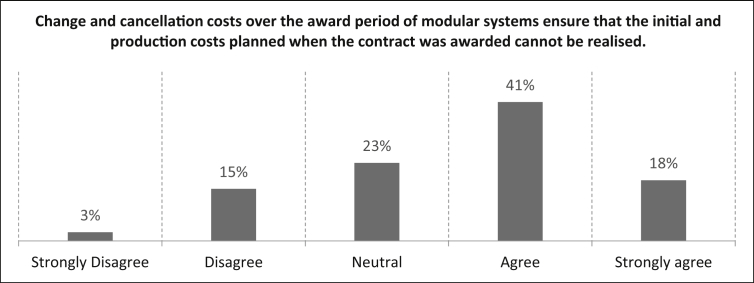
Evaluation of change and cancellation costs over the award period.

Change and cancellation costs reduce company profits, as they have a negative impact on the margins of the affected products each time they occur [3,7]. Because several products in the manufacturing industry are based on modular systems [8] it is necessary to reduce change and cancellation costs to a minimum. In Fig. 11, participants are asked for their degree of agreement with the statement *“Change and cancellation costs over the award period of modular systems ensure that the initial and production costs planned when the contract was awarded cannot be realised”*.

**Fig. 12:**
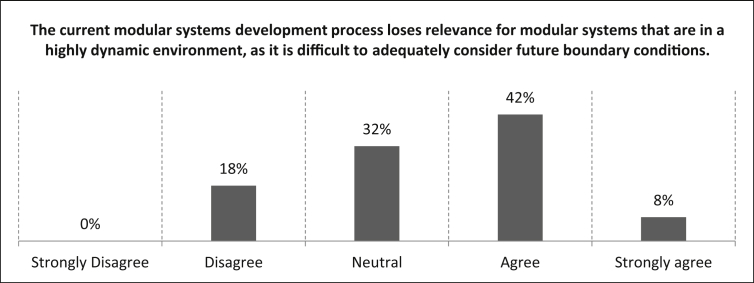
Evaluation of necessity of a new development process for modular systems in a highly dynamic environment.

All of the following statements focus on the challenges of modular systems in a highly dynamic environment. In Fig. 12 participants are confronted with the statement *“The current modular systems development process loses relevance for modular systems that are in a highly dynamic environment, as it is therefore difficult to adequately consider future boundary conditions”*.

The answers show that 42% of the participants agree and eight percent strongly agree with the presented statement. A share of 32% of the participants rated the statement neutrally and 18% disagree. The number of samples for this statement is n = 38.

**Fig. 13:**
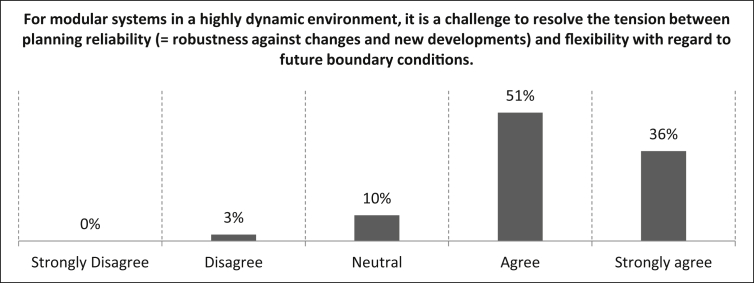
Rating of the solution of the conflict between planning reliability and flexibility with regard to future boundary conditions.

Subsequently, the proposition *“For modular systems in a highly dynamic environment, it is a challenge to resolve the tension between planning reliability (*= *robustness against changes and new developments) and flexibility with regard to future boundary conditions”* was presented. As the data in Fig. 13 shows, the vast majority of the participants (87%) agree or strongly agree with the statement. Modular systems in a highly dynamic environment have to be designed to be lasting and robust [2].

In the next question, the causal categories for the difficulties of reacting to future boundary conditions for modular systems in a highly dynamic environment are analysed.

**Fig. 14:**
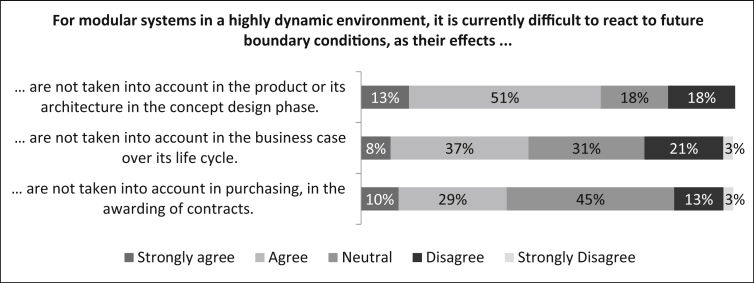
Reasons for difficulty in reacting to future boundary conditions for modular systems in a highly dynamic environment.

First they were asked if *“For modular systems in a highly dynamic environment, it is currently difficult to react to future boundary conditions, as their effects are not taken into account in the product or its architecture in the concept design phase.”* In Fig. 14 it can be seen that the majority (64%) of the survey participants agree or strongly agree with this statement. The remaining 36% are equally split up between the statement neutrally and disagree. Second they were asked if *“For modular systems in a highly dynamic environment, it is currently difficult to react to future boundary conditions, as their effects are not taken into account in the business case over its life cycle.”* Eight percent of participants strongly agree with this statement, 37% agree, 31% rate this statement neutrally and 24% disagree (3% strongly). Third they were asked if *“For modular systems in a highly dynamic environment, it is currently difficult to react to future boundary conditions, as their effects are not taken into account in purchasing, in the awarding of contracts.”* It can be seen that 39% of the participants agree or strongly agree with this statement. 45% rate this statement neutrally, 13% disagree and only three percent strongly disagree.

**Fig. 15:**
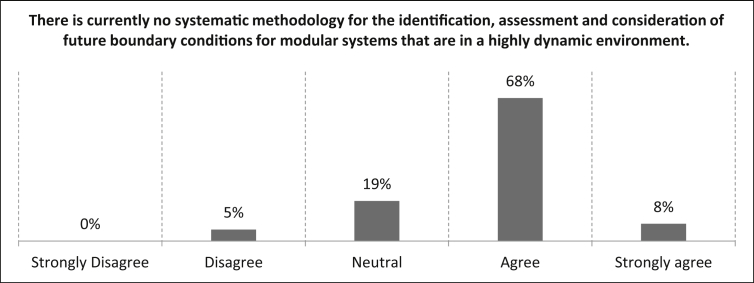
Rating of the necessity of a systematic methodology for the evaluation and consideration for modular systems that are in a highly dynamic environment.

In Fig. 15, the participants are asked whether there currently is a need for a systematic methodology for the identification, assessment and consideration of future boundary conditions for modular systems in a highly dynamic environment. The majority (76%) of the participants agreed with this proposal, while 19% gave a neutral assessment and five percent disagreed. The number of samples for this statement is n = 37.

**Fig. 16:**
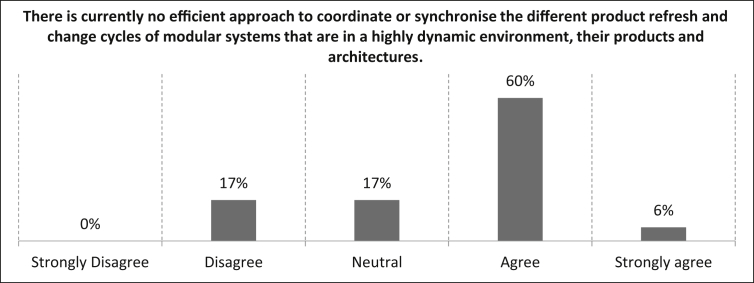
Evaluation of the approach to coordinate or synchronise the different life and change cycles of modular systems that are in a highly dynamic environment, their products and architectures.

Next, the proposition *“There is currently no efficient approach to coordinate or synchronise the different life and change cycles of modular systems that are in a highly dynamic environment, their products and architectures.”* was presented and the participants are asked for their opinion on this statement. It can be derived from Fig. 16, that 66% of the participants agree (six percent strongly) with the statement. The remaining 34% can be divided equally between a neutral and disagree assessment. The number of samples for this statement is n = 35.

**Fig. 17:**
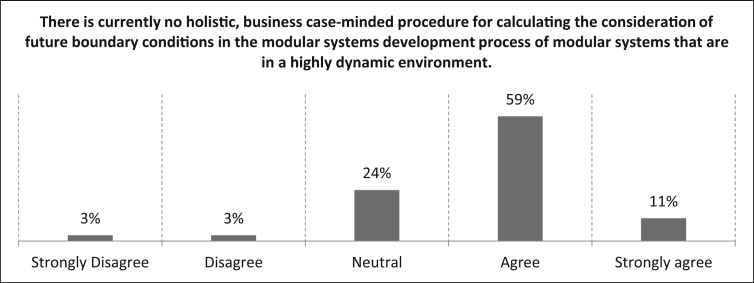
Rating of the necessity of a holistic, business case-minded procedure for calculating the consideration of future boundary conditions in the modular system development process of modular systems that are in a highly dynamic environment.

In the last question of the second part the participants are asked for their degree of agreement with the statement *“There is currently no holistic, business case-minded procedure for calculating the consideration of future boundary conditions in the modular systems development process of modular systems that are in a highly dynamic environment.”* With an agreement of 70% (59% agree and eleven percent strongly agree), the participants confirm the statement below. 24% gave a neutral assessment, three percent disagreed and three percent strongly disagreed. The number of samples for the statement of Fig. 17 is n = 37.

### 2.2 Survey 2 – modular systems dealing with innovations

#### 2.2.1 Experimental design, materials, and methods

##### 2.2.1.1 Presentation of the survey sample

**Fig. 18:**
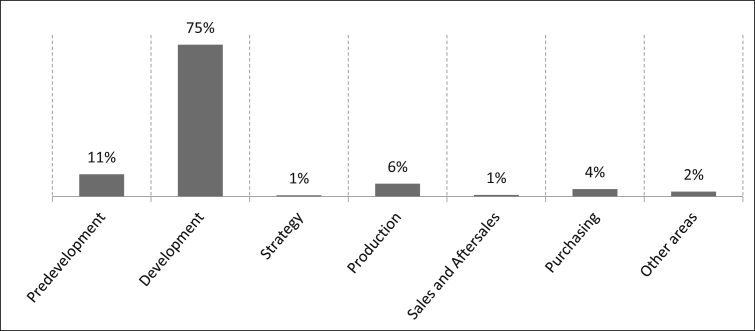
Business areas of the staff.

The first part of the questionnaire is intended to characterise the sample of the survey. As the data in Fig. 18 shows all of the 501 participants work for German automotive manufacturers. The business areas in which the survey participants work are as follows: 11% of the participants work in predevelopment, 75% in development, one percent in strategy, six percent in production, one percent in sales and after-sales and four percent in purchasing. Another two percent are listed under the category “Other areas” and an example of this would be finance. The number of samples is n = 490.

**Fig. 19:**
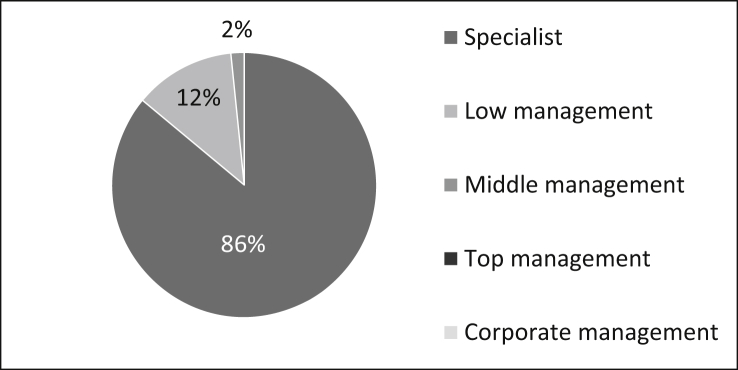
Roles of the staff.

The roles of the survey participants are manifold, as shown in Fig. 19: The majority .(86%) of the survey participants are specialists, 13% are active in lower management and two percent in middle management. There are no participants from the top and corporate management. The number of samples is n = 494.

**Fig. 20:**
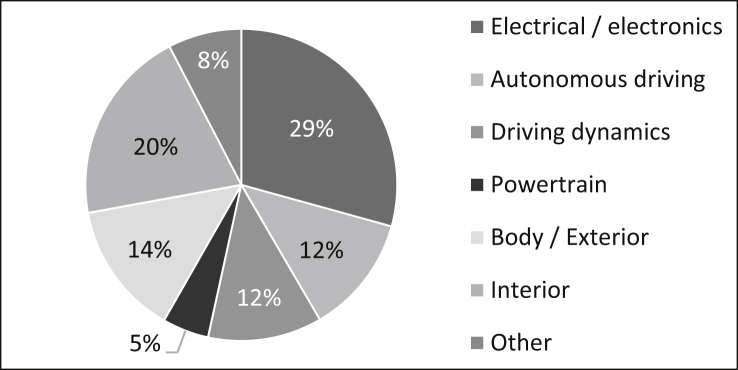
Subject areas of the staff.

As can be seen in Fig. 20 the survey participants come from the following areas: 29% work in the area of electrical/electronics, twelve percent in the area of autonomous driving, twelve percent in the area of driving dynamics, five percent in the area of powertrain, 14% in the area of body/exterior, 20% in the area of interior and eight percent in other areas such as design. The number of samples is n = 642 due to the possibility of multiple answers.

**Fig. 21:**
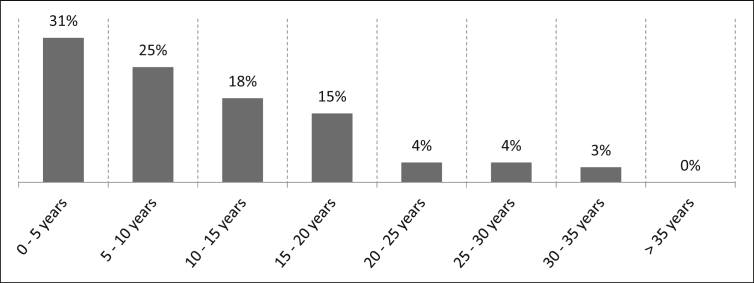
Length of service with the company.

The participants' length of service with their company are as follows: 31% of the participants have been with their company for 0–5 years, 25% for 5–10 years, 18% for 10–15 years, 15% for 15–20 years, four percent for 20–25 years, another four percent for 25–30 years and three percent for 30–35 years. There are no participants with a length of service with their company of over 35 years (See Fig. 21). The number of samples is n = 495.

##### 2.2.1.2 Current situation of modular systems in dealing with innovations

In the second part of the survey, participants are confronted with various statements about whether and where companies see challenges posed by modular systems. Further statements refer in particular to the current situation and the challenges for modular systems in the realization of innovations. In this context, the temporal and content-related focus of this survey will be established. The survey focuses on stage 2 of the standard stage gate process according to COOPER [9]. This phase is called “Build Business Case” [10] and focuses on the implementation of customer-oriented product content that corresponds to the goals of the business case to be achieved. Within the framework of customer-oriented product content, this study focuses only on innovations. For this reason, the focus in terms of content is on integrating innovations into the development process of the modular system as a kind of pre-development project and preliminary stage of the actual development in phase 3.

**Fig. 22:**
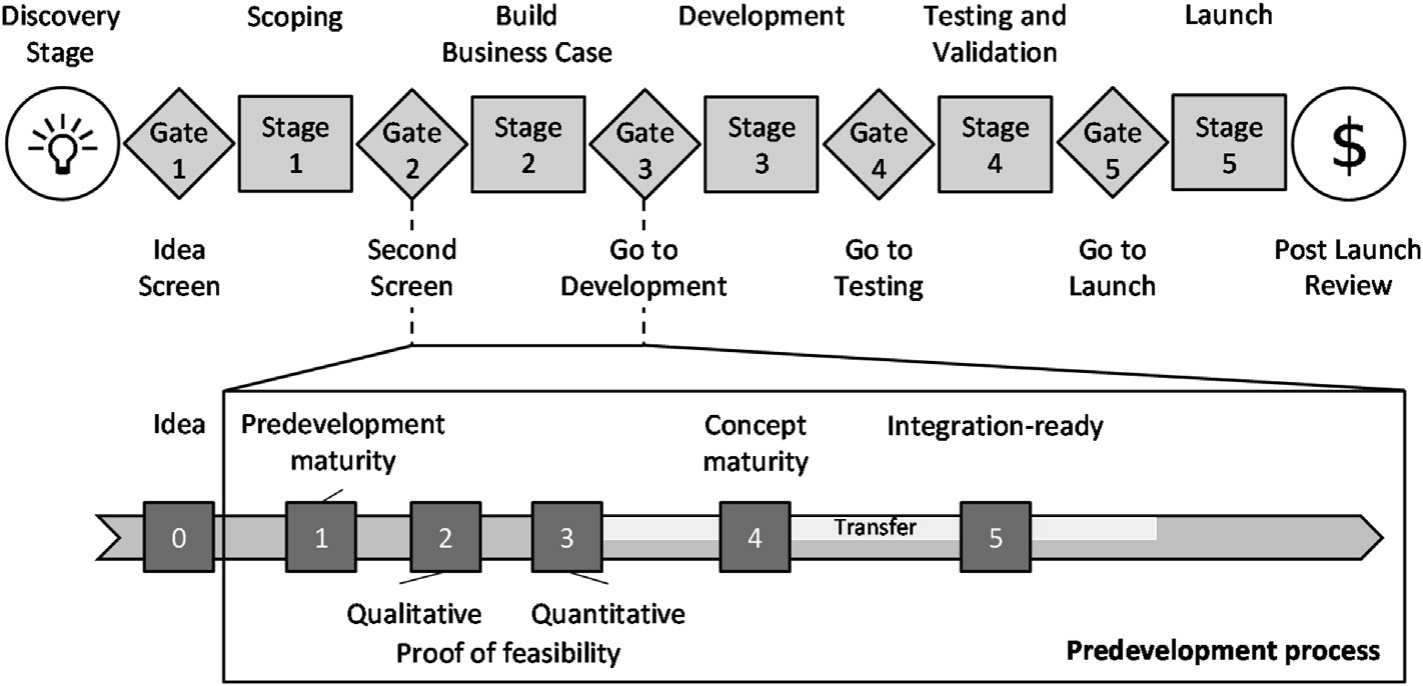
Temporal and content-related focus of this study [7].

On the basis of maturity level management, JAHN has defined six degrees of maturity that support an efficient and targeted transfer of innovations into the development process of modular systems at an early stage, as can be seen in Fig. 22. With the help of the continuous maturity assessment of the innovations, an increase in the success rates of the realized innovations is to be achieved, especially in the development times over several years [10]. The degree of maturity 0 lies in the scoping phase before gate 2. The other five degrees of maturity can be assigned to the “Build Business Case” phase. Innovations with a degree of maturity 4 or 5 already have been assigned to implementation in a product, which is characterized by a transfer process. For each of the six degrees of maturity, there are a few set goals that must be met in order to move on, as shown below [10]:

• Degree of maturity 0 “Idea”: The project idea and the goals are described and evaluated with regard to feasibility, customer benefits and the market potential.
• Degree of maturity 1 “Predevelopment maturity”: The basic technological and economic feasibility is presented. A recommendation on the part of the department for predevelopment is available. Technical killer criteria have been listed.
• Degree of maturity 2 “Proof of feasibility - Qualitative”: A solution for the function/property is identified and evaluated (technically and economically). Current requirements regarding the target product line are met. First prototype samples are available.
• Degree of maturity 3 “Proof of feasibility - Quantitative”: The technology is controllable and the integration risk is known and assessable. Quantified statements about e.g. operating strengths and costs can be made.
• Degree of maturity 4 “Concept maturity”: The technical implementation and integration risk, business aspects, affordability, and resource planning are roughly assessed by the responsible departments.
• Degree of maturity 5 “Integration-ready”: The technical implementation and integration risk, business aspects, affordability and resource planning are assessed in detail by the responsible departments.

At each degree of maturity of the innovation process, different parameters are taken into account that represent the progress of the project. In this study, five criteria were defined on the basis of which a statement can be made about the feasibility at the respective levels. Due to similar goals of the described maturity levels, the levels 0 and 1, levels 2 and 3 and levels 4 and 5 were combined in order to keep the response effort for the participants low.

**Fig. 23:**
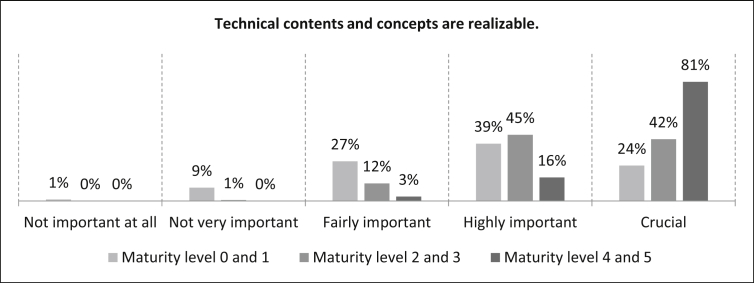
Technical contents and concepts criterion.

First the participants were asked how important it is that “Technical contents and concepts are realizable” at the different levels of maturity. Fig. 23 shows that for the maturity levels 0 and 1, 24% of the participants consider this statement to be crucial. 39% rate this statement as highly important, 27% as fairly important and ten percent as not important (one percent as not important at all). The number of samples is n = 499. For maturity levels 2 and 3, 42% of the participants rate the statement as crucial and 45% as highly important. The fairly important rating is twelve percent and only a small minority of one percent does not consider this statement to be very important. The number of samples is n = 496. At maturity levels 4 and 5, the vast majority (81%) of the respondents agree and rate the this statement as crucial. 16% rate this statement as highly important and three percent as fairly important. The number of samples is n = 499.

**Fig. 24:**
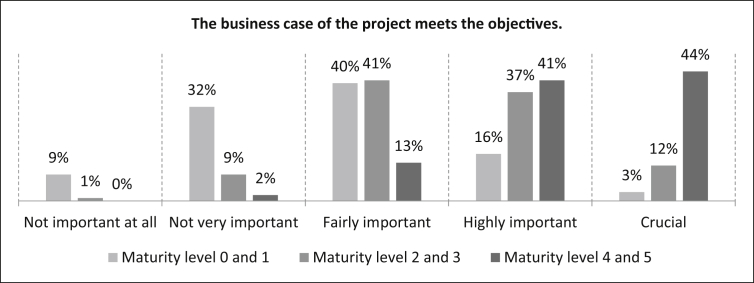
Business case criterion.

Secondly, the respondents were asked how important it is that the *“The business case of the project meets the objectives”* at the different levels of maturity. In the maturity levels 0 and 1, three percent of the participants rate the statement as crucial, 16% as highly important, 40% as fairly important, 32% as not very important and nine percent as not important at all. The number of samples is n = 498. In the maturity levels 2 and 3, the crucial rating rises up to twelve percent. The statement is rated as highly important by 37%, fairly important by 41%. Ten percent rated it as not important (one percent as not important at all). The number of samples is n = 498. At the maturity levels 4 and 5, 44% of the participants rate this statement as crucial, 41% as highly important, 13% as fairly important and only two percent as not very important. The number of samples is n = 495 (See Fig. 24).

**Fig. 25:**
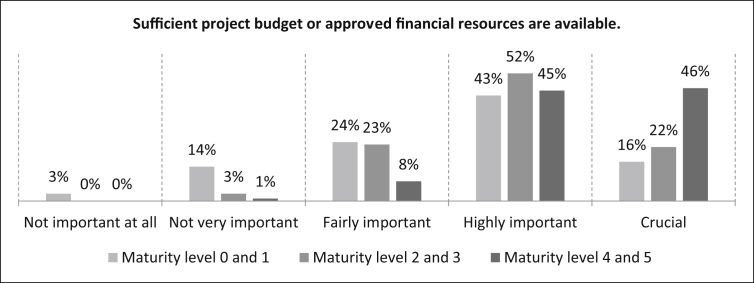
Project budget criterion.

The participants are then asked for their degree of agreement with the statement “S*ufficient project budget or approved financial resources are available”* for the different maturity levels. With a percentage of 16% the participants rate the statement in Fig. 25 as crucial for the maturity levels 0 and 1. In this context the highly important evaluation is 43%, and 24% of the participants rate this statement as fairly important. The statement is rated by 14% of the participants as not very important and by three percent not important at all. The number of samples is n = 495. For maturity levels 2 and 3, 22% of the participants rate this statement as crucial, 52% as highly important, 23% as fairly important and three percent as not very important. The number of samples is n = 495. For maturity levels 4 and 5, the evaluation shows that 46% consider this statement to be crucial and 45% to be highly important. Eight percent rate it as fairly important and only one percent as not very important. The number of samples is n = 498.

**Fig. 26:**
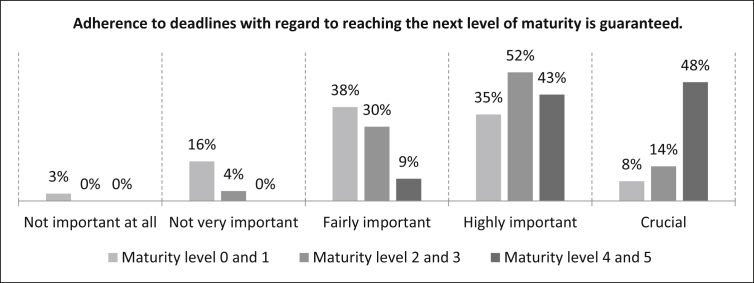
Adherence to deadlines criterion.

The participants were then asked how important it is that *“Adherence to deadlines with regard to reaching the next level of maturity is guaranteed” f*or the different levels of maturity. For the maturity levels 0 and 1, eight percent of the participants rate the statement as crucial, 35% as highly important, 38% as fairly important, 16% as not very important and three percent as not important at all. The number of samples is n = 498. For the maturity levels 2 and 3 there is a 14% rating for a crucial evaluation. 52% rate the statement as highly important, 30% as fairly important. The remaining four percent go to the not very important rating. The number of samples is n = 496. The statement is considered as crucial by 48% for maturity levels 4 and 5, and by 43% as highly important. The fairly important rating is at nine percent. The number of samples is n = 499 as can be seen in Fig. 26.

**Fig. 27:**
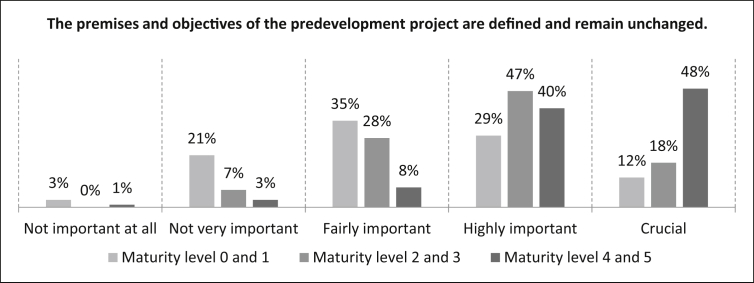
Premises and objectives criterion.

In Fig. 27, participants are asked how important it is that *“the premises and objectives of the predevelopment project have been defined and remain unchanged.”* For the maturity levels 0 and 1 it can be seen that twelve percent of the participants rate the statement as crucial, 29% as highly important, 35% as fairly important, 21% as not very important and three percent as not important at all. The number of samples is n = 499. Eighteen percent rate the statement as crucial for maturity levels 2 and 3. The statement is rated by 47% of the participants as highly important and by 28% as fairly important. Only seven percent consider the statement as not very important. The number of samples is n = 496. For the maturity levels 4 and 5 there is a 48% crucial rating on the statement. Also 40% rate the statement as highly important, eight percent as fairly important and three percent as not very important. The number of samples is n = 497.

All of the following statements concentrate on the innovation drivers and the associated urgency of implementation in modular systems. In practice, it has been observed that entry level products are rarely first-time users of innovations through pre-development projects [11]. For this reason, we also distinguish between pre-development projects and the diffusion of innovations. The term diffusion of innovation encompasses the entire process of market penetration by new products and services driven by social influences. This includes all interdependencies between consumers that influence the various market participants with or without their explicit knowledge. Diffusion thus includes the general dissemination of an innovation in the market [11].

In order to be able to assess the urgency of implementing innovations in modular systems and to compare pre-development projects with the diffusion of innovations, we have identified two internal and three external innovation drivers for modular systems [[12], [13], [14]]:

- Internal urgency: Contribution to the fulfilment of the corporate strategy; customer feedback shows that there is a lack of innovation in a certain business area.
- External urgency: Anticipated legislative changes; new consumer protection requirements; the existence of innovations in certain business areas by direct competitors.

**Fig. 28:**
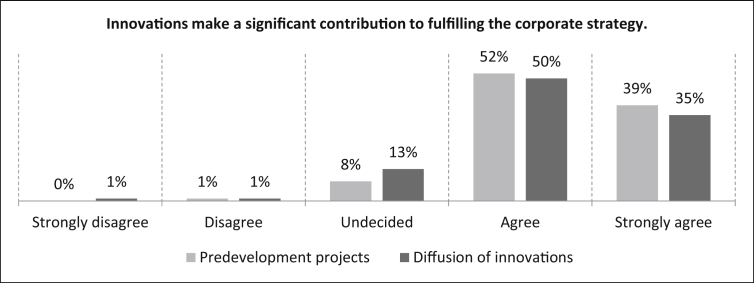
Contribution to the fulfilment of the corporate strategy – Internal urgency.

First, we start with innovation drivers that can be assigned to the internal urgency. For this reason we asked the participants whether *“innovations make a significant contribution to fulfilling the corporate strategy”*. For predevelopment projects, Fig. 28 shows that the majority of respondents (91%) agree or strongly agree with this statement. The remaining nine percent are divided into an undecided rating (eight percent) and one percent disagree on the statement. The number of samples is n = 496. A majority (85%) of participants agree (35% strongly) with the statement. There are 13% of the participants who are undecided about this statement and only one percent who disagree. The number of samples is n = 480.

**Fig. 29:**
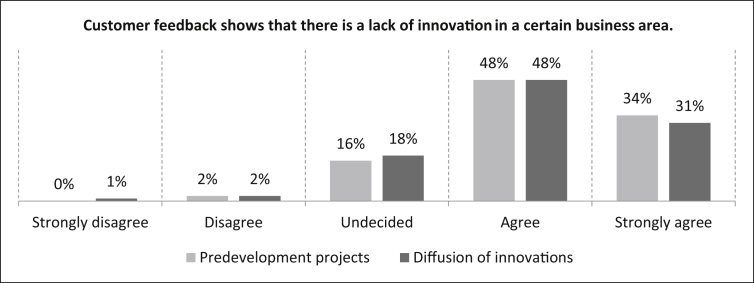
Customer feedback - Internal urgency.

In Fig. 29, participants are asked whether *“customer feedback shows that there is a lack of innovation in a specific field of the company”* as part of the internal urgency. For predevelopment projects, the answers show that 48% of the participants agree and 34% strongly agree with the statement presented. The minority of the participants (16%) rate the statement as undecided and two percent disagree. The number of samples is n = 495. A share of 31% of the respondents strongly agree and 48% agree with the statement regarding the diffusion of innovations. There are 18% of the participants who are undecided and three percent who disagree (one percent strongly). The number of samples is n = 478.

**Fig. 30:**
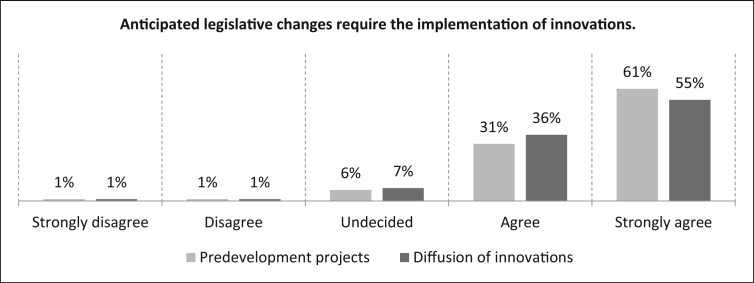
Anticipated legislative changes - External urgency.

The upcoming three statements can be assigned to the external urgency. In this regard, the next question confronts the participants with the statement that *“anticipated legislative changes require the implementation of innovations”*. In predevelopment projects, 61% of participants strongly agree with the statement, 31% agree, six percent consider it undecided and two percent disagree (one percent strongly). The number of samples is n = 496. For the diffusion of innovations, 91% of the respondents agree with the statement, 55% strongly. Seven percent gave an undecided assessment and the remaining two percent split equally between disagreement and strong disagreement with the statement. The number of samples is n = 476 (See Fig. 30).

**Fig. 31:**
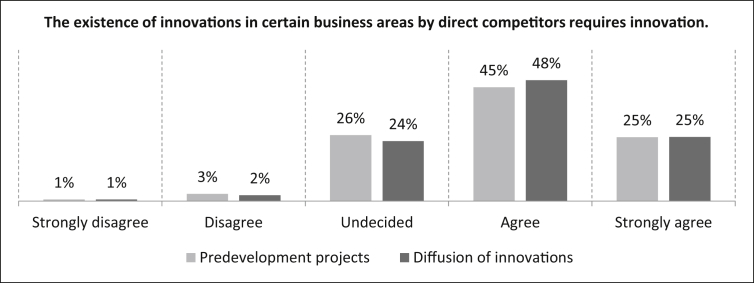
Innovations in certain areas by direct competitors - External urgency.

Next comes the statement *“The existence of innovations in certain business areas by direct competitors requires innovation”* as part of the external urgency. For the predevelopment projects, Fig. 31 shows that 25% of the participants strongly agree, 45% agree and 26% are undecided about the statement. Three percent of the participants disagree and one percent strongly disagree. The number of samples is n = 494. For the diffusion of innovations, 25% of the respondents also strongly agree with the statement. 48% of the participants agree, 24% are undecided about the statement and three percent disagree (one percent strongly). The number of samples is n = 480.

**Fig. 32:**
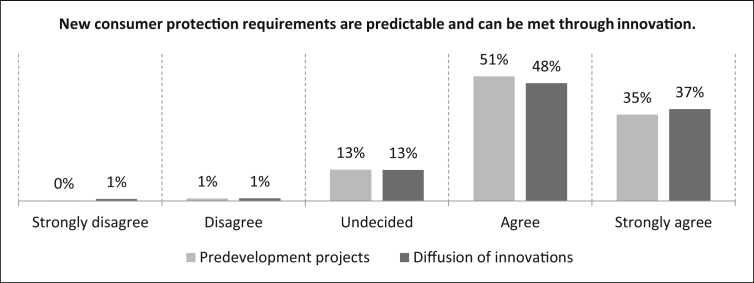
New consumer protection requirements - External urgency.

In the last question of the second part, the participants are asked about their degree of agreement with the statement *“New consumer protection requirements are predictable and can be met through innovation”* as part of the external urgency. For predevelopment projects it can be seen that 86% of the participants agree with the statement (35% strongly). A further 13% are undecided about the statement and one percent disagree. The number of samples is n = 494. For the diffusion of innovation it can be observed in Fig. 32, that 37% of the respondents strongly agree with the statement, 48% agree and 13% are undecided. One percent of the participants disagree and another one percent strongly disagree. The number of samples is n = 475.
